# The role of soils in the regulation of ocean acidification

**DOI:** 10.1098/rstb.2020.0174

**Published:** 2021-09-27

**Authors:** P. Renforth, J. S. Campbell

**Affiliations:** The Research Centre for Carbon Solutions, Heriot-Watt University, Edinburgh EH14 4AS, UK

**Keywords:** ocean alkalinity, enhanced weathering, ocean acidification

## Abstract

Soils play an important role in mediating chemical weathering reactions and carbon transfer from the land to the ocean. Proposals to increase the contribution of alkalinity to the oceans through ‘enhanced weathering’ as a means to help prevent climate change are gaining increasing attention. This would augment the existing connection between the biogeochemical function of soils and alkalinity levels in the ocean. The feasibility of enhanced weathering depends on the combined influence of what minerals are added to soils, the formation of secondary minerals in soils and the drainage regime, and the partial pressure of respired CO_2_ around the dissolving mineral. Increasing the alkalinity levels in the ocean through enhanced weathering could help to ameliorate the effects of ocean acidification in two ways. First, enhanced weathering would slightly elevate the pH of drainage waters, and the receiving coastal waters. The elevated pH would result in an increase in carbonate mineral saturation states, and a partial reversal in the effects of elevated CO_2_. Second, the increase in alkalinity would help to replenish the ocean's buffering capacity by maintaining the ‘Revelle Factor’, making the oceans more resilient to further CO_2_ emissions. However, there is limited research on the downstream and oceanic impacts of enhanced weathering on which to base deployment decisions.

This article is part of the theme issue ‘The role of soils in delivering Nature's Contributions to People’.

## 1. Introduction

The Earth's climate is regulated by processes on the land and ocean. Soils play an important role in both spheres as a medium for organic carbon accumulation and turnover. Soils also facilitate mineral weathering, which removes CO_2_ from the atmosphere, converts it into bicarbonate ions, which contribute to the alkalinity of the ocean. This relationship between terrestrial and oceanic processes is an important feature in the natural carbon cycle [1], specifically as a feedback balancing volcanic degassing and other natural CO_2_ accumulation in the atmosphere. Weathering will also consume all anthropogenic CO_2_ emissions over 10^3^–10^6^ years [2,3].

The role of soils is particularly relevant to ‘enhanced weathering’ proposals that consider adding minerals to the land to help mitigate climate change [4,5]. Every year, the Earth's rivers naturally add around 500 million tonnes of dissolved calcium to the oceans [6]. This calcium originates from the weathering of carbonate or silicate minerals, which (along with other cations: Mg, Na and K) also consumes CO_2_ (e.g. equations (1.1) and (1.2)).

1.1 $${\mathrm{CaCO}}_{3(s)}+{\mathrm{CO}}_{2(g)}+H_{2}O_{\left(l\right)}\to {\mathrm{Ca}}_{\left(\mathrm{aq}\right)}^{2+}+2{\mathrm{HCO}}_{3(\mathrm{aq})}^{-}$$

and

1.2 $${\mathrm{CaSiO}}_{3(s)}+2{\mathrm{CO}}_{2(g)}+3H_{2}O_{\left(l\right)}\to {\mathrm{Ca}}_{\left(\mathrm{aq}\right)}^{2+}+2{\mathrm{HCO}}_{3(\mathrm{aq})}^{-}+H_{4}{\mathrm{SiO}}_{4(\mathrm{aq})}$$

Equations (1.1) and (1.2) show the reaction of single minerals (calcite CaCO_3_ and wollastonite CaSiO_3_) with CO_2_, but typically a range of minerals in a rock weather to produce an array of dissolved species as well as new mineral phases (including clay minerals and iron oxides) but, as above, typically consume CO_2_ [4]. Approximately 0.25 billion tonnes (Gt) of carbon (1 GtC = 1 peta gram C) may be removed from the atmosphere by natural weathering of silicate minerals [7–9], and a similar amount from carbonate weathering [10]. On geological timescales, this removal is balanced with CO_2_ emissions from volcanic sources. Changes in this balance are fundamental in the climate system, and the temperature dependence of weathering rate provides a long-term negative feedback, stabilizing global climate [11].

Soils play an important role as a medium in which weathering reactions take place [12]. Mineral weathering is naturally accelerated in soils through physical (freeze–thaw, wetting–drying and anthropogenic activities [13]) and biochemical (CO_2_ respiration, and proton/organic molecule exudation from plant roots, microbes and fungal hyphae, [14,15]) processes. Being composed mainly of secondary minerals (minerals that form through environmental processes, see below, e.g. clays, carbonates and iron oxides/hydroxides), soils are also a product of weathering, which may occlude fresh primary minerals in underlying rock and reduce further mineral dissolution [16]. Soil is also a medium for the reverse reaction of equation (1.1), in which ‘pedogenic’ carbonates are formed. The quantification of the carbonate content of soil has typically been confined to arid environments where it is the largest carbon pool [17]. Estimates suggest that 695–748 GtC are stored globally as pedogenic carbonate, in which the calcium is derived primarily from remobilised lithogenic carbonate [18].

Here, the fundamental role that soils play in the terrestrial-oceanic inorganic carbon cycle are explored, and how, through the action of soils, enhanced weathering may help to ameliorate ocean acidification.

## 2. The role of soils in enhanced weathering

The application of crushed carbonate minerals to soils is a standard practice in agriculture (agricultural lime) to amend soil porewater pH. It is likely that in excess of 100 Mt of agricultural lime (CaCO_3_) are applied globally (e.g. 20–30 Mt in the US alone [19], although global figures are not readily available). By mimicking natural weathering, but using similar processes and supply chains for agricultural lime, some have suggested the intentional addition of silicate minerals to the land surface may help to prevent climate change [20–23], with the additional consequence of increasing ocean alkalinity [24]. Enhanced weathering may be part of a portfolio of approaches that intend to remove multiple GtCO_2_ yr^−1^ from the atmosphere by 2100 [25,26]. For instance, a recent study suggests that the application of crushed basalt to 35–59% of cropland area in 12 countries could be sufficient to remove 2 GtCO_2_ yr^−1^ by 2050 at a cost of $60–220 per tCO_2_ [5]. The technical challenges associated with enhanced weathering are dominated by the need to crush rock to a small particle size, such that the rate of mineral dissolution is sufficiently rapid that a large proportion of the mineral dissolves over only a few years. As such, the costs in Beerling *et al*. [5] account for emissions produced by the supply chain. Below we consider the properties of, and processes in, soils that may control the function of enhanced weathering. Rather than adding silicate minerals, it is theoretically possible to add carbonate minerals to the land surface (e.g. expanding the use of agricultural lime). However, as we discuss below, such a proposal may be considerably limited in the CO_2_ removed per unit of land.

The inorganic components of soil are conceptually divided into primary and secondary minerals. Primary minerals have not been significantly chemically altered since their crystallization from molten material [27]. They are mainly silicate minerals with varied bonding structure [28]. Other common primary minerals in soils include oxides/hydroxides of titanium/iron/manganese, carbonates, as well as non-crystalline inorganic materials such as volcanic glasses [29]. Primary minerals undergo various physical, chemical, biochemical and human-induced weathering in soils. One of the main weathering pathways is the reaction with natural aqueous solutions, such as rainwater, where carbonic acid forms by dissolution of atmospheric CO_2_. Carbonic acid reacts with the surfaces of primary minerals causing them to dissolve. On short timescales, weathering of carbonate minerals (e.g. equation (1.1)) results in less net sequestration of CO_2_ than weathering of silicate minerals (e.g. equation (1.2)), and that, over longer timescales (over hundreds of thousands to millions of years), weathering of carbonates results in no net CO_2_ sequestration due to eventual re-precipitation of carbonates in the ocean [4].

In soils, the CO_2_ partial pressure may be between 10 to 100 times greater than that of the atmosphere due to plant and microbial respiration, bringing it into the same range as power station flue gas [30,31]. This elevated partial pressure generates additional acidity, accelerating mineral weathering. Moreover, weathering in soils is enhanced by the release of organic acids from plant roots, e.g. malic and acetic acid [32,33], microorganisms, e.g. fulvic, humic, phenolic acids [34,35] and fungi, e.g. citric and oxalic acid [36,37]. In addition, organic compounds can form complexes with the cations in silicate minerals, facilitating breakdown as well as altering the formed products [38]. Furthermore, earthworms, lauded by Aristotle as ‘the intestines of the earth’, play a significant role in enhancing mineral degradation, via organic acids, digestive enzymes and gut microbes during ingestion as well as via burrow aeration and transport processes [39,40].

Since most weathering occurs via contact between primary minerals and aqueous solutions, mineral solubility is important. Generally, silicate minerals with less silica polymerization, e.g. olivine, dissolve at faster rates than minerals with greater silica polymerization, e.g. quartz [41,42] owing to the stronger Si–O bond compared to the M–O bond (where M = Na, Mg or Ca, etc). The dissolution of carbonate minerals (equation (1.1)) is orders of magnitude fasted than silicate minerals, and carbonate dissolution is congruent, meaning the molar ratios of the dissolved elements in solution are similar to that of the solid. However, most primary silicate minerals dissolve incongruently, which means their more soluble components are released preferentially [28]. For instance, when in contact with natural waters, minerals tend to release monovalent cations (e.g. Na^+^, K^+^), before divalent cations (Mg^2+^, Ca^2+^), before trivalent cations (Fe^3+^, Al^3+^), according to the correlation between the ease of hydrolysis and electrostatic valency of the species [43].

In soils, the dissolved products of primary silicate mineral weathering increase the availability of some limiting nutrients such as Si, K and P [44]. These can boost plant productivity and increase the size of the terrestrial carbon pool [45]. This process is critical in natural soil formation [46]. Some of the dissolved products, namely bicarbonate HCO_3_^−^, are transported by rivers to the oceans, increasing its total alkalinity, and counteracting ocean acidification (see below [47]). Furthermore, dissolved Si, P and Fe could stimulate biological productivity in oceans, removing additional CO_2_ from the atmosphere as organic carbon [48–50].

Alongside production of bioavailable dissolved products, incongruent dissolution of some primary minerals also produces solid residues, referred to as secondary minerals. For example, during weathering, primary mineral feldspars, MAlSi_3_O_8_, hydrolyse, releasing soluble cations M^+^ and H_4_SiO_4_, and leaving behind the solid secondary (clay) mineral kaolinite, Al_2_Si_2_O_5_(OH)_4_ (e.g. equation (2.1)).

2.1 $$2{\mathrm{KAlSi}}_{3}O_{8(s)}+9H_{2}O+2H_{\left(\mathrm{aq}\right)}^{+}\to {\mathrm{Al}}_{2}{\mathrm{Si}}_{2}O_{5}{\left(\mathrm{OH}\right)}_{4(s)}+4H_{4}{\mathrm{SiO}}_{4(\mathrm{aq})}+2K_{\left(\mathrm{aq}\right)}^{+}$$

Other common secondary minerals in soils include oxides, e.g. Fe_2_O_3_, hydroxides, e.g. Al(OH)_3_, carbonates, e.g. CaCO_3_, and phosphates, e.g. Ca_5_(PO_4_)_3_(F, Cl, OH).H_2_O. Secondary minerals may also precipitate directly from aqueous solution rather than by continuous modification of a primary mineral [51]. The compositions, structures and quantities of these secondary minerals together with organic molecules determine a soils' cation exchange capacity (CEC) and thus its ability to hold nutrients and buffer against acidification [52]. Although clays are more stable to weathering than the primary minerals from which they are derived, they too undergo weathering. In tropical soils, where temperature and precipitation are high, and where decaying organic matter is plentiful, clays undergo additional breakdown [28]. For example, kaolinite may hydrolyse, forming gibbsite (Al_2_O_3_.3H_2_O):

2.2 $${\mathrm{Al}}_{2}{\mathrm{Si}}_{2}O_{5}{\left(\mathrm{OH}\right)}_{4(s)}+5H_{2}O_{\left(l\right)}\to {\mathrm{Al}}_{2}O_{3}.3H_{2}O_{\left(s\right)}+2H_{4}{\mathrm{SiO}}_{4(\mathrm{aq})}$$

Field and laboratory studies [53–57] have shown that clay formation can significantly limit the extent and rate of primary mineral weathering and control elemental fluxes [53,58]. There are primarily three ways in which the precipitation of clays moderate dissolution rates of primary minerals: (i) via control of the saturation state of primary minerals in natural waters; (ii) forming passivating coatings on primary minerals restricting their reactive surface area; and (iii) reducing the hydraulic conductivity of the soil and/or creating preferential flow channels [57].

Another major factor in soil weathering is the presence of the transition metals Fe and Mn and their related redox processes [59]. In primary minerals, Fe and Mn mainly occur in their reduced form, i.e. Fe(II) and Mn(II). Their oxidation creates a charge imbalance which destabilizes the mineral lattice, enabling weathering [28]. In addition, the acidity created by oxidation in aqueous environments facilitates further mineral breakdown (equation (2.3)).

2.3 $$2{\mathrm{Fe}}_{\left(\mathrm{aq}\right)}^{2+}+0.5O_{2(g)}+3H_{2}O_{\left(l\right)}\to 2{\mathrm{FeOOH}}_{\left(s\right)}+4H_{\left(\mathrm{aq}\right)}^{+}$$

The global organic carbon content of soils is roughly three times more than that of atmospheric or terrestrial biomass [60] and a small perturbation to this pool can have a dramatic effect on atmospheric CO_2_ concentrations [61,62]. Secondary minerals play a very large role in the stabilization and retention of soil organic matter [63]. Secondary minerals form micro- and macro-aggregates with organic matter creating a physical barrier against attacking microbes [64–69]. Soil organic matter can also become stabilized by chemical or physicochemical binding with secondary minerals to form organomineral complexes [70,71]. Without these protections, organic carbon would decompose and mineralize, entering the atmosphere, and eventually result in acidification of the oceans [72].

As such, soil pore water chemistry is fundamental to enhanced weathering, while the ‘carrying capacity’ of rainwater, soil porewaters, and runoff may be constrained by secondary mineral formation. For instance, table 1 considers the metal cation concentration (Mg^2+^ or Ca^2+^) and dissolved inorganic carbon (DIC) of a solution in equilibrium with a range of primary and secondary minerals and 400 µatm of CO_2_ (approximately the partial pressure of CO_2_ in the atmosphere), and 50 000 µatm of CO_2_ (a typical partial pressure of CO_2_ in soil pore gases). The total alkalinity varies by over 8 orders of magnitude depending on what minerals are dissolving or precipitating, and the partial pressure of CO_2_. An effective enhanced weathering strategy may require spatial removal on the order of 10's tCO_2_ ha^−1^ yr^−1^ [5], which is thermodynamically possible for most silicate minerals at 50 000 µatm CO_2_, but only for a smaller selection of primary/secondary mineral pairs at 400 µatm CO_2_. Thus, the feasibility of enhanced weathering depends on the combined influence of dissolving primarily minerals, the formation of secondary minerals, and the partial pressure of CO_2_. These determine the maximum possible flux of basic cations to oceans via river transport and thus the transport of alkalinity to the ocean.

**Table 1.** Resulting (*a*) total dissolved inorganic carbon and (*b*) total alkalinity from geochemical equilibrium between primary dissolving minerals (rows) and secondary precipitating minerals (columns). (*c*) The conversion of DIC to spatial flux assuming 500 mm rainfall. Calculated using PHREEQC [73] and the LLNL.dat database file (apart from gehlenite, which was calculated using minteq.dat file).

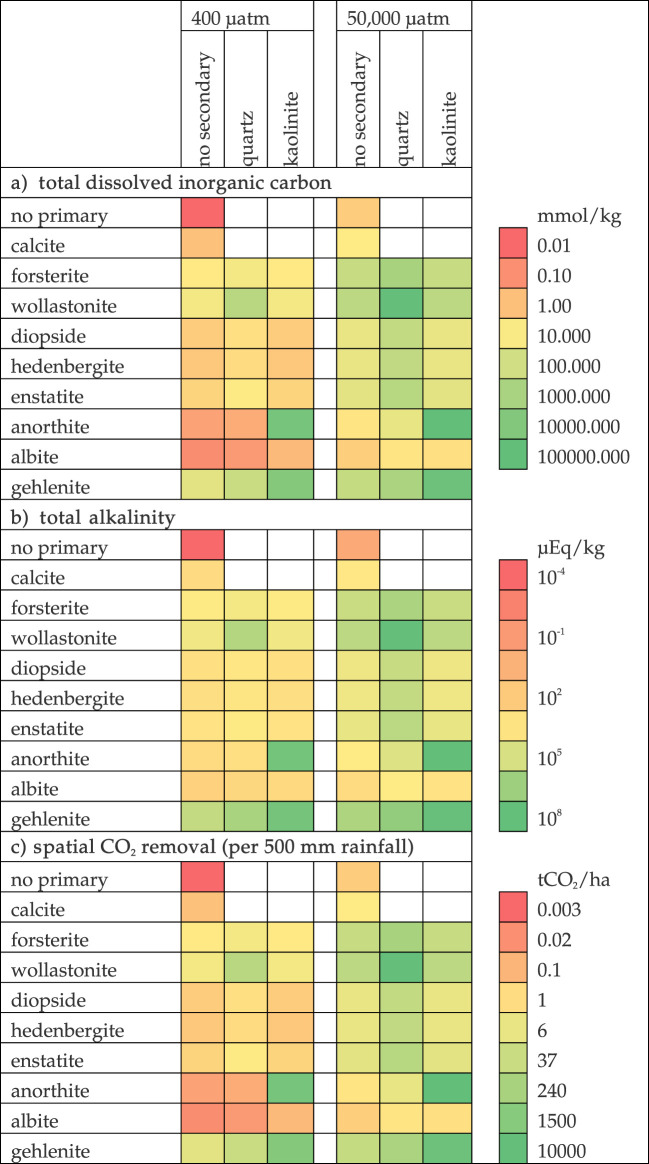

Table 1 also highlights the limitations of using calcite, the mineral in agricultural lime, within enhanced weathering strategies. Here a spatial CO_2_ draw-down of 0.1–1 tCO_2_ ha^−1^ is 1–2 orders of magnitude smaller than what might be possible with silicate minerals. However, calcite may dissolve orders of magnitude faster than some silicate minerals, which may result in lower processing requirements and potentially cheaper removal costs. Its effectiveness as a CO_2_ removal technology may be constrained if the intention is large CO_2_ removal over a definite land area. However, it still may be possible to dissolve carbonate minerals within engineered systems where the produced alkaline solutions are added to the ocean [74].

Agricultural activities can substantially enhance mineral weathering and the flux of alkalinity to the oceans. For example, tillage exposes less-weathered minerals at depth and brings them to the surface where weathering rates are faster. Acidification resulting from application of fertilisers may also enhance mineral dissolution [75–77]. Nitrification of nitrogen-rich fertilisers can create nitric acid, HNO_3_, which reacts with minerals (equation (2.4)) at rates exceeding that of natural carbonic acid.

2.4 $$2{\mathrm{KAlSi}}_{3}O_{8(s)}+9H_{2}O_{\left(l\right)}+2{\mathrm{HNO}}_{3(\mathrm{aq})}\to {\mathrm{Al}}_{2}{\mathrm{Si}}_{2}O_{5}{\left(\mathrm{OH}\right)}_{4(s)}+4H_{4}{\mathrm{SiO}}_{4(\mathrm{aq})}+2K_{\left(\mathrm{aq}\right)}^{+}+2{\mathrm{NO}}_{3(\mathrm{aq})}^{-}$$

However, the role of nitrification in mediating weathering has previously been thought not to result in sequestration of atmospheric CO_2_, and in the case of carbonate weathering could promote CO_2_ emission [75,77–79]. Similarly, sulphur deposition (e.g. dissolved into rainwater), water acidification through oxidation of sulphur-bearing minerals (e.g. acid mine drainage), could promote weathering while resulting in the emission of CO_2_ [80].

Research on natural and enhanced weathering suggests that soils have an important influence on the generation of alkalinity which is ultimately transported to the oceans. This alkalinity influences the oceanic carbon cycle and the ability of the oceans to take up CO_2_.

## 3. The ocean carbon cycle and acidification

**Figure 1.**
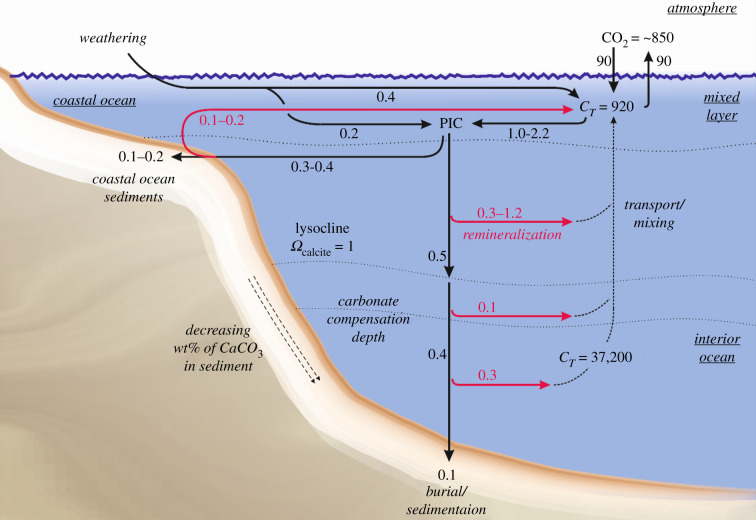
The global ocean carbonate cycle. Adapted from Andersson and Sabine & Tanhua [6,82]. Arrows represent fluxes in Gt C per year (red arrows denote remineralization). *C_T_* represents the dissolved inorganic carbon pools in Gt C. PIC, particulate inorganic carbon.

The ocean is the largest carbon pool at the Earth's surface containing approximately 40 000 GtC. This includes organic carbon contained within living biomass (3 GtC) and dissolved organic carbon (700 GtC). Molecules within the carbonate system, namely aqueous carbon dioxide (CO_2(aq)_), bicarbonate ions (HCO_3_^−^) and carbonate ions (CO_3_^2−^) comprise the majority of oceanic carbon, of which approximately 920 GtC resides in surface waters and 37 200 GtC in the deep ocean [81]. Figure 1 presents a schematic of the oceanic inorganic ‘carbonate’ cycle, in which all 850 Gt of atmospheric C is cycled through DIC (*C*_*T*_ in figure 1) within a decade. Marine autotrophic organisms consume DIC to produce biomass, but some calcifiers (e.g. corals, coccolithophores) also use this carbon to form mineral carbonate shells [83], which ultimately becomes particulate inorganic carbon (PIC). Note that unlike autotrophy, carbonate shell formation consumes ocean alkalinity and generates CO_2_/acid (reverse of equation (1.1). Much of the PIC is remineralized back into CO_2_, HCO_3_^−^ and CO_3_^2−^ as it sinks into corrosive deeper waters (or through biological mediated weathering in the surface ocean) with only a minor amount (approx. 0.3 GtC yr^–1^) reaching the ocean floor and being permanently removed as sediment [6].

The ease by which organisms create mineral carbonate shells is related to the product of the activity of the dissolved constituents (here Ca^2+^ and CO_3_^2−^) normalized to mineral solubility (equation (3.1), [84,85]). The activity of calcium in seawater is relatively stable. However, CO_3_^2−^ ions are in dynamic equilibrium with CO_2_ in seawater, such that its activity is reduced by elevated aqueous CO_2_ (equation (3.2)).

3.1 $$\Omega =\;\frac{\alpha {\mathrm{Ca}}^{2+}\;\cdot \;\alpha {\mathrm{CO}}_{3}^{2-}}{K_{\mathrm{sp}}}$$

and

3.2 $${\mathrm{CO}}_{2\;(\mathrm{aq})}+H_{2}O⇌{\mathrm{HCO}}_{3}{\;}^{-}+H^{+}\;⇌{\mathrm{CO}}_{3}{\;}^{2-}+2H^{+}.$$

The ocean has absorbed nearly 40% of anthropogenic CO_2_ emissions since the industrial revolution [86], and subsequently depressed the saturation state of the carbonate mineral aragonite (CaCO_3_) (referred to as ‘ocean acidification’ [84]). This process can be represented by equation (3.3), in which CO_3_^2−^ ions are consumed through reaction with CO_2_ to produce HCO_3_^−^ (thus decreasing Ω). This places stress on marine calcifying organisms, some of which are sensitive to these changes [87,88] and additional acidification caused by contemporary and future emissions may have severe impacts on some ecosystems. Taylor *et al*. [89] suggest that an enhanced weathering scheme may be able to counteract the changes caused by saturation state through a globally deployed enhanced weathering scheme. However, the protection offered to calcifying organisms may be geographically limited to regions in which enhanced weathering is deployed.

3.3 $${\mathrm{CO}}_{2}+{\mathrm{CO}}_{3}{\;}^{2-}⇌\;2{\mathrm{HCO}}_{3}{\;}^{-}.$$

Research over the last 20 years to understand the impact of ocean acidification [84] has produced variable results [90,91]. Species that can maintain calcium carbonate saturation levels in their internal calcifying sites may be less affected by changes in seawater pH. However, elevated CO_2_ will force calcifying organisms to expend a greater amount of energy in shell building, which could have the largest impact on sensitive organisms/ecosystems (e.g. some corals [92,93]), and some marine environments may dip below safe calcium carbonate saturation levels by mid-century [94].

By reacting away aqueous CO_2_ (equation (3.3)) additional CO_2_ can be removed from the atmosphere. This buffering capacity was formalised by Revelle & Suess [95], into what has subsequently been termed the ‘Revelle Factor’ (RF, equation (3.4)).

**Figure 2.**
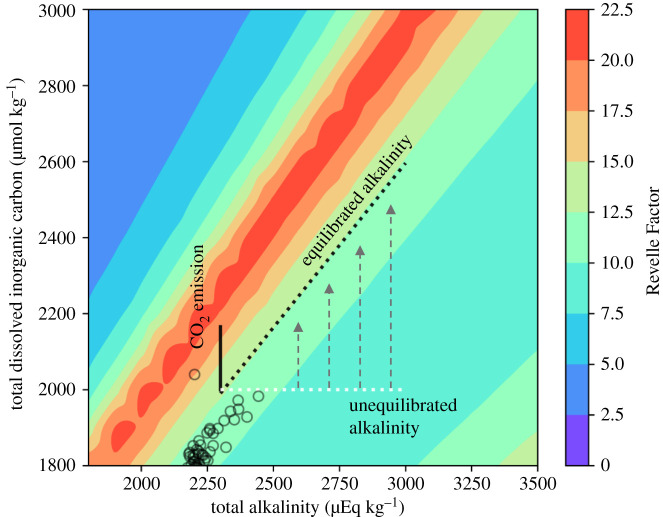
The Revelle Factor (derived from Egleston *et al*. [96]) for values of total dissolved inorganic carbon and total alkalinity. The open circles show estimated values over the past 2 million years (derived from Hönisch [97]). The lines show stylised trajectories of an RCP6.0 magnitude emissions scenario (see Renforth & Henderson [24]) that is unabated (solid line) or wholly mitigated by enhanced weathering (dotted lines). (Online version in colour.)

3.4 $$RF=\;\frac{\partial \ln[{\mathrm{CO}}_{2}]}{\partial \ln\mathrm{DIC}}$$

where the partial differentials denote that other state variables (e.g. total alkalinity) are held constant. RF describes the respective change of DIC with changes in atmospheric *p*CO_2_. A larger RF equates to a reduction in oceanic buffering capacity. Figure 2 shows a projection of RF under an RCP6.0 type emissions scenario, in which current oceanic values have already diverted from preindustrial and will continue to increase over the coming century, equating to a reduction in the buffering capacity by approximately 34% between 2000 and 2100 [98].

Figure 2 also shows that it may be possible to maintain an RF value of the surface ocean if all anthropogenic CO_2_ emissions were mitigated by increasing ocean alkalinity (e.g. through mineral weathering). Initially, when alkalinity is increased it will not be equilibrated with atmospheric CO_2_ (figure 2, white line) and the RF would be reduced. Following the equilibration with CO_2_, the RF value would be maintained for a given emission. While mitigating all anthropogenic emissions by increasing ocean alkalinity is unlikely to be technically possible or desirable, this hypothetical exercise illustrates that enhanced weathering may also help to maintain the CO_2_ buffering capacity of the ocean.

As in the case of the re-precipitation of carbonate minerals via marine calcification, the ‘reverse weathering’ of silicate minerals in seawater can also occur. This involves the combination of dissolved metal anions, silicic acid, aluminium hydroxide and bicarbonate, to precipitate cation-poor clay minerals and generate CO_2_, which may be released to the atmosphere. Such reactions play an important role in controlling the global geochemical balance [99]. They remove alkalinity from the ocean and control the partitioning of CO_2_ in the ocean atmosphere system. For example, the formation of saponite, Ca_0.15_Na_0.1_Mg_2.5_Fe_0.8_Si_3_AlO_10_(OH)_2_ (equation (3.5)):

3.5 $$0.15{\mathrm{Ca}}^{2+}+0.1{\mathrm{Na}}^{+}+2.5{\mathrm{Mg}}^{+}+0.8{\mathrm{Fe}}^{+}+3H_{4}{\mathrm{SiO}}_{4}+\mathrm{Al}{\left(\mathrm{OH}\right)}_{3}+7{\mathrm{HCO}}_{3}{\;}^{-}\to {\mathrm{Ca}}_{0.15}{\mathrm{Na}}_{0.1}{\mathrm{Mg}}_{2.5}{\mathrm{Fe}}_{0.8}{\mathrm{Si}}_{3}{\mathrm{AlO}}_{10}{\left(\mathrm{OH}\right)}_{2}+7{\mathrm{CO}}_{2}+10H_{2}O.$$

The source of Si may also be biogenic opal and the source of Al may be ‘degraded clays’ [100]. Reverse weathering reactions can also involve reactions with solids, e.g. FeOOH-rich coatings on substrate grains [100]. Reactions such as the one in equation (3.5) primarily occur in marine and deltaic environments. For example, *in situ* clay formation has been observed in the Amazon and Mississippi river deltas [53]. However, it has also been found to occur in the closed-basin lakes of Ethiopia [101]. The extent to which reverse weathering occurs at hydrothermal vents is subject to debate [102–104].

Michalopolous & Aller [53] determined that reverse weathering reactions in Amazon shelf sediments could consume as much as 10% of the continental riverine K^+^ flux. However, the true extent of reverse weathering is difficult to quantity due to the small quantities of clays formed in addition to interference from terrestrially derived clays [100]. Thus, the process is poorly understood, and its contribution remains uncertain [105]. A range of clay minerals are formed in marine environments, including greenalite, minnesotaite, palygorskite, montmorillonite, glauconite, berthierine, chamosite, clinochlore, sudoite, odenite and corrensite. Of these, only greenalite and minnesotaite are thought to be formed exclusively in marine environments and may be used to help distinguish between marine and terrestrial sources [105]. Better understanding of reverse weathering has been made possible using isotope tracking, particularly K [106], Li [107], and more recently Be [108].

Although formation of clays via reverse weathering is thermodynamically favoured, they may be spatially and kinetically constrained owing to a silica limitation [105]. Indeed, it is postulated that the late ecological rise of siliceous organisms and the resulting decline in silica-rich conditions inhibited the rate of reverse silicate mineral weathering, causing higher ocean alkalinity and lower atmospheric CO_2_ levels [105]. This silica limitation on reverse weathering has been observed in experiments in the Amazon delta [53]. On the other hand, supply of Al and/or Fe were kinetic limiters in clay formation in the Mississippi delta [109]. Reverse weathering reactions generate CO_2_ and consume seawater alkalinity. As such, the relative rates of these processes could affect the efficacy of using ocean alkalinity enhancement as an atmospheric CO_2_ management strategy and as a way of helping chemically counter ocean acidification [110]. For instance, saponite formation has been reported during olivine dissolution experiments in a laboratory shaker [111] and vermiculite and saponite were observed in flume weathering studies [112]. Formation of these clays reduces the efficiency of ocean alkalinity enhancement, e.g. coastal enhanced weathering of olivine which aims to sequester CO_2_ as bicarbonate in the ocean [113].

## 4. Conclusion

Sustainable Development Goal 14 aims to ‘conserve and sustainably use the oceans, sea and marine resources for sustainable development’, with a target to ‘minimize and address the impacts of ocean acidification, including through enhanced scientific cooperation at all levels'. The most effective approach to prevent impacts of ocean warming, acidification and sea level rise on SDG 14 is to stabilize if not reduce atmospheric CO_2_ concentrations by quickly moving to net-zero CO_2_ emissions. This requires both a redoubled effort to dramatically reduce CO_2_ emissions as well as employing methods to pro-actively remove billions of tonnes per year of atmospheric CO_2_.

In the context of this required CO_2_ removal, we have outlined the significant role soils already play in the land–ocean carbon cycle, and how safely accelerating chemical transformations here could contribute to CO_2_ removal efforts as well as help rebalance ocean chemistry. Reactions mediated in soils including respiration and organic acid exudation can accelerate weathering rates, while secondary mineral formation and elevated CO_2_ may limit the maximum alkalinity flux to the ocean. This alkalinity contributes to removing CO_2_ from the atmosphere for storage as DIC in the ocean.

Secondary mineral formation depends on the type of primary mineral dissolving as well as the local climate and biota present. CO_2_ sequestration potential of enhanced weathering in soils will need to be determined by *in situ* monitoring with special care to characterize the secondary minerals formed in order to accurately determine the total amount of carbon removed from that atmosphere.

The oceans remove approximately 25% of anthropogenic CO_2_ emissions, and some of this excess CO_2_ is neutralised through carbonate buffering (i.e. the ‘Revelle Factor’). The remaining CO_2_ contributes to a reduction in ocean pH, which will decline further if CO_2_ emissions continue to rise. This buffering capacity in the ocean has diminished as a consequence of reaction with CO_2_, and will continue to reduce with additional CO_2_ emissions, thus reducing the amount of CO_2_ that can be removed and neutralised. By increasing the alkalinity flux from land to the ocean, via enhanced weathering schemes, it may be possible to partially replenish the buffering capacity of the ocean and ameliorate some of the impacts of ocean acidification.

## Data accessibility

This article has no additional data.
